# Efficacy and safety of PD-1 inhibitors plus anti-angiogenesis tyrosine kinase inhibitors with or without transarterial chemo(embolization) for unresectable hepatocellular carcinoma: a meta-analysis

**DOI:** 10.3389/fonc.2024.1364345

**Published:** 2024-08-22

**Authors:** Yue Chen, Luyao Jia, Yu Li, Wenhao Cui, Jukun Wang, Chao Zhang, Chunjing Bian, Tao Luo

**Affiliations:** ^1^ Department of General Surgery, Xuanwu Hospital, Capital Medical University, Beijing, China; ^2^ Emergency Medicine Department, Xuanwu Hospital, Capital Medical University, Beijing, China

**Keywords:** hepatic artery infusion chemotherapy, transarterial chemoembolization, tyrosine kinase inhibitors, PD-1 inhibitors, unresectable hepatocellular carcinoma

## Abstract

**Background:**

The triple combination of programmed cell death protein–1 (PD-1) inhibitors plus anti-angiogenesis tyrosine kinase inhibitors (TKIs) with or without transarterial chemoembolization (TACE) or hepatic arterial infusion chemotherapy (HAIC) enhance the effect of treatment for unresectable hepatocellular carcinoma (uHCC). The present study compared the efficacy and safety of PD-1 plus TKI with or without transarterial chemo(embolization) for uHCC.

**Methods:**

The meta-analysis was conducted using data acquired from PubMed, EMBASE, the Cochrane Library, Ovid, Web of Science, and Clinical Trials.gov from the inception date to December 2023. All clinical outcomes of interest included overall survival (OS), progression-free survival (PFS), objective response rate (ORR), and adverse events (AEs). The hazard ratio (HR) and risk ratio (RR) with 95% confidence intervals (CIs) were used to measure the pooled effect. In addition, subgroup analysis was conducted to determine the specific patient population that benefited.

**Results:**

The OS (HR = 0.47; 95% CI: 0.39–0.56, *P < * 0.05), PFS (HR = 0.52; 95% CI: 0.45–0.60, *P < *0.05), and ORR (RR = 1.94; 95% CI: 1.60–2.35, *P <* 0.05) were significantly better in TACE/HAIC+TKI+PD-1(TACE/HAIC TP) group than TKI+PD-1(TP) group. The incidence of AEs was acceptable.

**Conclusion:**

The triple therapy of TACE/HAIC TP had better efficacy for uHCC than TP, with acceptable security.

**Systematic review registration:**

PROSPERO, identifier CRD42023475953.

## 1 Introduction

Hepatocellular carcinoma (HCC) is known as a leading cause of cancer-related mortality globally. The incidence and mortality of HCC still remain high, of which primary liver cancer is the most common (1, 2). The majority of patients with HCC lose the opportunity for surgery, ablation, and liver transplantation, as they are in the intermediate and late stage when diagnosed, resulting in a poor prognosis (3). So far, there is no clear guideline recommending the best treatment regime for the so-called unresectable hepatocellular carcinoma (uHCC).

According to the Barcelona Clinic Liver Cancer (BCLC) treatment strategy or Chinese National Liver Cancer (CNLC), transarterial chemoembolization (TACE) is the first globally recognized as the preferred treatment method for uHCC patients, but it also has certain limitations (4). Recently, hepatic artery infusion chemotherapy (HAIC) is usually applied for uHCC, especially for patients with poor response to TACE (5, 6). In Japan, HAIC has been recommended in treatment guidelines for HCC (7). For advanced-stage HCC, Sorafenib, a multi-kinase inhibitor, was approved be the first-line treatment by the U.S. Food and Drug Administration (FDA) in 2007 (8). In the first line of tyrosine kinase inhibitors (TKIs) in 2008, Lenvatinib was found to be non-inferior to Sorafenib for HCC (9).

Recently, immune checkpoint inhibitors (ICIs), particularly programmed death protein–1 (PD-1) inhibitors, were shown to be clinically beneficial for patients with advanced HCC, leading to more options for uHCC. The clinical trials demonstrated the combination therapy with lenvatinib and the PD-1 inhibitor nivolumab resulted in better OS and ORR in patients with intermediate or advanced-stage HCC (10). The IMbrave150 trial, the ORIENT-32 trial, and the Phase III trial of camrelizumab plus apatinib revealed that ICIs combined with anti-angiogenesis TKI also significantly improved the outcomes in patients with advanced HCC than the previous first-line treatment of TKI alone (11, 12). Therefore, new systemic therapy is the preferred option for uHCC patients currently; there is growing evidence that PD-1 inhibitors and anti-angiogenesis TKI in combination are recommended as systemic treatments in uHCC patients at present (13, 14). The reason is that multi-kinase inhibitor with potent antiangiogenic properties, which can reverse the immunosuppressive microenvironment of tumors and enhance the immune anti-tumor efficacy of PD-1. In addition, numerous clinical trials have evaluated that TKI+PD-1 can improve HAIC/TACE-induced hypoxia and modulate the immunosuppressive microenvironment of uHCC (15, 16). Nevertheless, the triple therapeutic strategies for uHCC are debatable, and the efficacy and safety of TACE/HAIC+ TKI +PD-1(TACE/HAIC TP) remain unclear.

TACE or HAIC are recommended as important treatments for uHCC. PD-1 plus anti-angiogenesis TKI has also resulted in satisfactory outcomes in the treatment of uHCC. Therefore, this meta-analysis was conducted to determine the efficacy and safety of TACE/HAIC + TKI +PD-1(TACE/HAIC TP) versus TKI +PD-1(TP) for uHCC.

## 2 Materials and methods

The article has been reported in line with the PRISMA (Preferred Reporting Items for Systematic Reviews and Meta Analyses) checklist (17). This meta-analysis was registered in PROSPERO (CRD42023475953).

### 2.1 Literature search strategy

**Figure 1:**
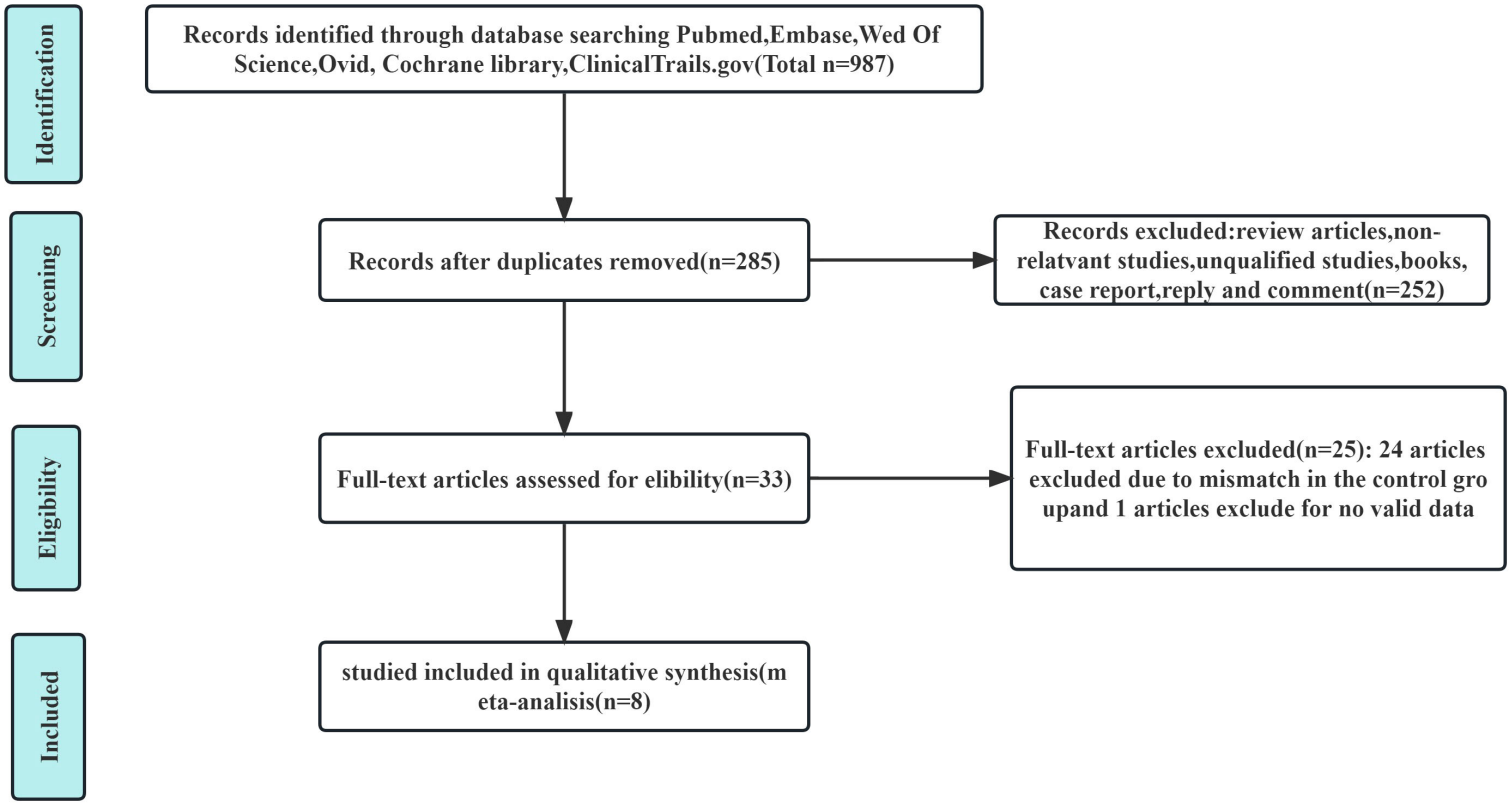
Flowchart of article screening and selection process.

The publication time was limited to when the databases were established until December 2023. We conducted a systematic search of PubMed, EMBASE, the Cochrane Library, Ovid, Web of Science, and ClinicalTrials.gov databases to identify useful literature related to this meta-analysis. The MESH terms used in these databases included (“carcinoma, hepatocellular” or “liver cancer” or “HCC” or “liver neoplasm”), [“hepatic arterial infusion chemotherapy” (HAIC) or “TACE”], (“PD-1” or “ICI”) (“anti-angiogenesis TKIs”). There were no language limitations or other restrictions imposed in the literature search strategy. Thereafter, two authors (X.X. and X.X.) independently extracted and confirmed relevant data. The flowchart of the article screening and selection process are presented in the **Figure 1**.

### 2.2 Study selection

#### 2.2.1 Inclusion criteria

1. The study patients with HCC in the intermediate and late stage according to BCLC and CNLC;
2. The patients with uHCC had received TACE/HAIC TP compared with TP;
3. The types of study include cohort studies and randomized controlled trials (RCTs);
4. The primary outcomes assessed were OS, PFS, ORR, and AEs, which include at least one evaluable survival outcome.

#### 2.2.2 Exclusion criteria

1. Unable to obtain full text of literature;
2. The study types included a review, a meta-analysis, a conference abstract, a letter, and a case report;
3. The study lacked effective outcomes data or reported irrelevant outcomes;
4. Other confounding factors.

### 2.3 Data extraction and quality assessment

Two reviewers (X.X. and X.X.) independently screened the studies and evaluated the quality of the included studies in a standardized way. Any discrepancy was resolved through a discussion, and a third reviewer (X.X.) would decide if necessary. The data extracted from each study include the name of the first author, the year of publication, the nationality and the design of the study population, and the clinical characteristics of patients (including sex, age, and HCC stage).

Version 2 of the Cochrane tool for assessing the risk of bias in randomized trials (RoB2) was applied for RCT and the Newcastle-Ottawa Scale (NOS) was applied for cohort studies (18, 19). The quality assessment of each literature is presented in **Table 1**.

**Table 1:** Baseline characteristics of the studies.

Study ID	Country	Design	Patients(M/F)		Age (years)		Child-Pugh classification(A/B)		ECOG(0/1/2)		BLCL(B/C)		Groups		NOS SCORE
			TACE/HAIC TP	TP	TACE/HAIC TP	TP	TACE/HAIC TP	TP	TACE/HAIC TP	TP	TACE/HAIC TP	TP	TACE/HAIC TP	TP	
Chen 2021	China	RCS	84 (72/12)	86 (71/15)	52 (42–67)	53 (43–69)	71/13	75/11	38/46/0	35/51/0	22/62	21/65	HAIC+L+Pembrolizumab	L+Pembrolizumab	9
Fu 2022	China	RCS	89 (83/6)	53 (50/3)	51.9	53.5	88/1	47/6	/	/	68/21	27/26	HAIC+L+P	L+P	8
Lang 2023	China	RCS	75 (66/9)	39 (34/5)	≤65 (76%)	≤65 (74.4%)	59/16	30/9	48/24/3	28/8/3	32/43	14/25	T+L+Sintilimab	L+Sintilimab	8
Mei 2021	China	RCS	45 (38/7)	25 (18/7)	49.1 ± 10.6	50.1 ± 12.3	44/1	22/3	/	/	5/40	3/22	HAIC+L+P	L+P	6
Xin 2023	China	RCS	60 (54/6)	58 (51/7)	57.5(26–76)	54.5 (28–78)	60/0	58/0	55/5/0	53/5/0	21/39	23/35	TACE+L+P	L+P	8
Li 2023	China	RCS	62 (55/7)	83 (71/12)	≥65 (19%)	≥65 (45%)	48/13	65/17	/	/	9/46	8/68	TACE+TKI+P	TKI+P	7
Guo 2022	China	RCS	31 (26/5)	23 (22/1)	≥60 (22.6%)	≥65 (47.8)	21/10	14/9	/	/	5/24	3/19	TACE+TKI+P	TKI+P	7
Huang 2022	China	RCS	24 (20/4)	40 (35/5)	58.0±10.7	57.8±13.1	18/16	28/12	11/13/0	17/23/0	/	/	TACE+TKI+P	TKI+I	8

RCS, retrospective cohort studies; M, male; F, female; HAIC, hepatic arterial infusion chemotherapy; TACE, transarterial chemoembolization; L, Lenvatinib; T, tyrosine kinase inhibitors; P, PD-1 inhibitors; ECOG, Electronics Coordinating Group; PS, Performance Status; BCLC, Barcelona Clinic Liver Cance.

## 3 Statistical analysis

Hazard ratios (HRs) with 95% confidence intervals (CIs) were calculated to analyze OS and PFS. The risk ratios (RRs) with 95% CIs were calculated to analyze ORR and AEs. A fixed effect model was used for data pooling if no significant heterogeneity among included trials was observed. Otherwise, a random effect model was used. Multiple subgroup analyses were conducted to investigate the possible sources of heterogeneity. The results of tests for trials are presented (*P* < 0.05 in the χ^2^ test suggested significant heterogeneity). The *I* ^2^ statistic values of <25%, 25%–50%, and >50% were considered low, moderate, and high heterogeneity, respectively. The *I* ^2^ statistic (*I^2^* > 50% was deemed to have significant heterogeneity) and chi-square test (*P* < 0.10 was deemed to suggest a significant heterogeneity) were used to assess the heterogeneity among the trials.

The funnel plots were performed to detect the existence of publication bias (*P* < 0.10 was deemed to represent significant publication bias). All analyses were performed using the Revman5.4 software.

## 4 Results

### 4.1 Search results

A total of eight articles satisfied the inclusion and exclusion criteria, of which 285 were selected after removing duplicates (20–27). After reviewing the titles and abstracts of 285 articles, we excluded 252 articles. The full text of the remaining 33 articles were evaluated. Then 25 studies were excluded for lacking an appropriate control group or valid data. Ultimately, eight articles were included in the current meta-analysis.

All selected articles are retrospective cohort studies without RCT. There are three experimental groups with HAIC+TKI+PD-1 (HAIC TP) and five experimental groups with TACE+TKI+PD-1 (TACE TP) (20–27), while all control groups with TP.

### 4.2 Study characteristics

The included study characteristics are summarized in **Table 1**. All included articles were published in China from 2021 to 2023. Among the 877 patients included in our study, 766 were males and 111 were females. Furthermore, 470 patients with uHCC received the triple therapy of TACE/HAIC TP, whereas 407 patients received TP. The dose and duration of HAIC/TACE TP regimens are shown in **Table 2**.

**Table 2:** The dose and duration of HAIC/TACE TP regimens.

Study ID	Chemotherapeutic agents	TKI agents	PD-1
Chen 2021	FOLFOX-HAIC: 85 mg/m2 oxaliplatin from hour 0 to 2 on day 1; 400 mg/m2 fluorouracil bolus at hour 3 and 2400 mg/m2 fluorouracil over 46 h on days 1 and 2; and 400 mg/m2 leucovorin from hour 2 to 3 on day 1	8–12 mg lenvatinib once daily	200mg Pembrolizumab once every 3weeks
Fu 2022	FOLFOX-HAIC: oxaliplatin 130 mg/m2, leucovorin 200 mg/m2, fluorouracil 400 mg/m2, and fluorouracil 2400 mg/m2 (46 h)	8–12 mg lenvatinib once daily	Pembrolizumab, Sintilimab, Toripalimab, Camrelizumab and Tislelizumab.
Lang 2023	TACE: 0.3 g of 300-500 µm microspheres ; 200 mL of a 300 mg diluted solution of carboplatin or lobaplatin	8 mg lenvatinib once daily	200 mg sintilimab once 3 weeks
Mei 2021	FOLFOX-HAIC: 85 or 135 mg/m2 oxaliplatin, 400 mg/m2 leucovorin and 400 mg/m2 fluorouracil on the first day; and 2400 mg/m2 fluorouracil over 46 h	8 mg lenvatinib once daily	100 mg Nivolumab; 200 mg Keytruda; 240 mg Toripalimab; 200 mg Sintilimab
Xin 2023	TACE: Oxaliplatin (75 mg/m2) and raltitrexed (3 mg/m2) or 5-fuorouracil (750 mg/m2); the emulsion of iodized oil (10–20 ml) mixed with chemotherapeutic drugs (epirubicin, 30–50 mg/m2 or pirarubicin, 20 mg/m2)	8–12 mg lenvatinib once daily	sintilimab, tislelizumab or camrelizumab
Li 2023	TACE: lipiodol emulsions (10–20 mL) and one or more chemotherapeutic drugs such as cisplatin, cisplatin and mitomycin C, or fluorouracil	TKI	PD-1
Guo 2022	TACE: iodized oil (5–20 mL) mixed with platinum (10–40 mg) or epirubicin (10–40 mg)	400mg Sorafenib (bid), 8 - 12 mg Lenvatinib or 250 mg Apatinib (daily)	200 mg camrelizumab once 3 weeks
Huang 2022	TACE: iodized oil (5–20 mL) mixed with platinum (10–40 mg) or epirubicin (10–40 mg)	800 mg Sorafenib (bid) or 8 - 12mg lenvatinib (daily)	camrelizumab or sintilimab (200 mg) every 3 weeks

Beneficiary populations are further identified through subgroup analyses based on the data of univariate analysis in each included trial.

### 4.3 Risk of bias

The results of the risk of bias analysis within studies are reported (**Table 1**). The methodological quality of the included studies was assessed using NOS as all included studies are retrospective studies. It contains the selection of subjects, comparability of the groups, and assessment of outcomes, with a maximum of 9 points. Studies with a score of more than 6 were determined to be high quality.

### 4.4 Meta-analysis outcomes

#### 4.4.1 Overall survival, progression-free survival, and objective response rate

**Figure 2:**
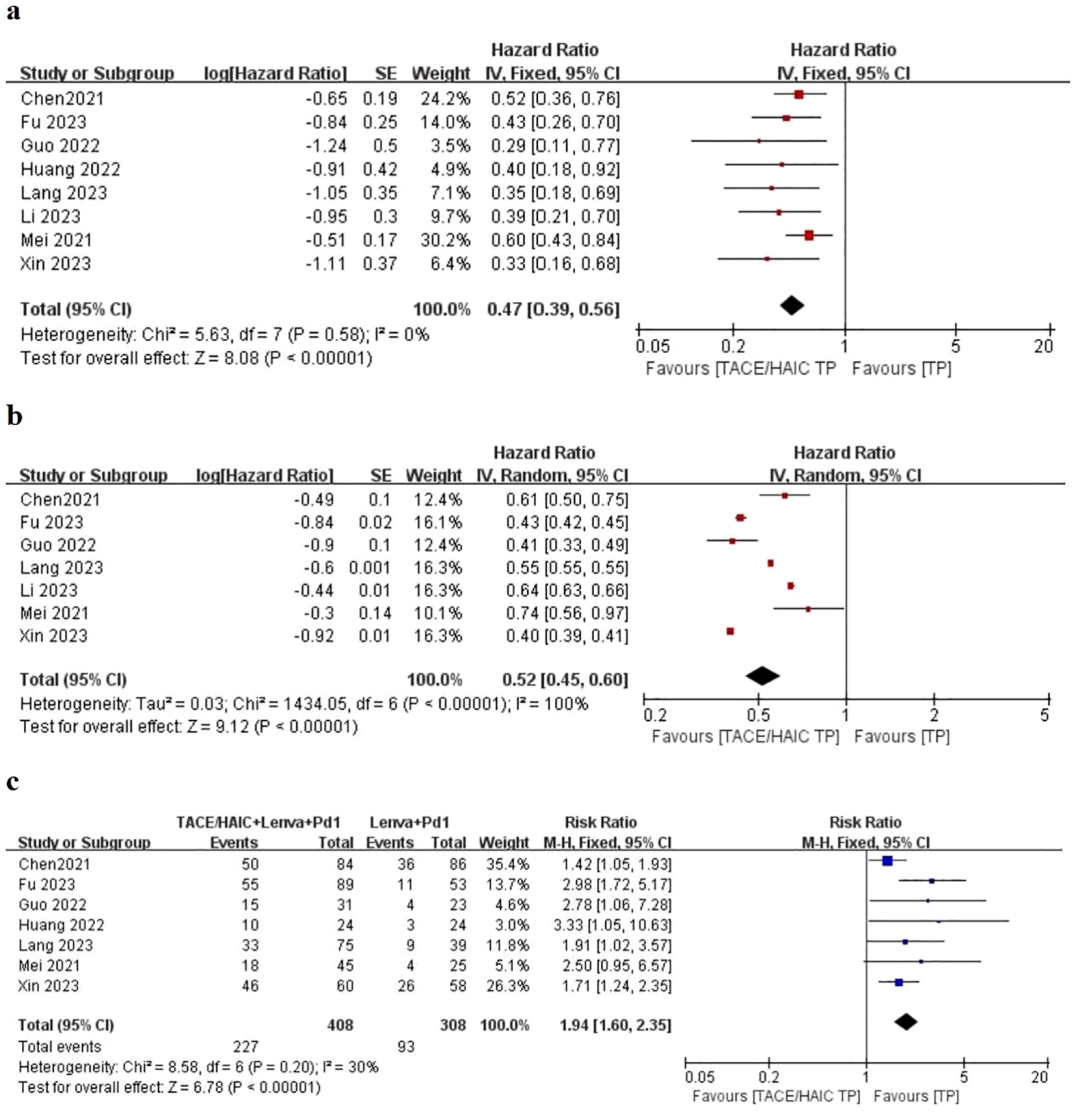
Forest plot about the pooled results of TACE/HAIC TP versus TP for unresectable HCC. Outcome: OS **(A)**, PFS **(B)**, and ORR **(C)** in total.

All eight articles in our study reported overall survival (OS) and PFS for the groups of TACE/HAIC TP and TP in **Figure 2**, including the point estimate (HR) and its 95% CI (17–24). Meta-analysis showed that triple therapy in the TACE/HAIC TP group had significantly longer OS (HR = 0.47; 95% CI: 0.39–0.56, *P < *0.05) and PFS (HR = 0.52; 95% CI: 0.45–0.60, *P < *0.05) than the TP group. As no significant heterogeneity was observed among the comparison of OS (*I* ^2^ = 0%, *P*  =  0.43), fixed effect models were adopted to estimate. While, for progression-free survival (PFS), significant heterogeneity was observed (*P* = 0.00001 < 0.1, *I^2^* = 100%), random effect models were adopted.

Patients in eight studies were assessed for ORR in **Figure 2**. The pooled analysis revealed that the objective response rate (ORR) (RR = 1.94; 95% CI: 1.60–2.35, *P <* 0.05) of the TACE/HAIC TP group was better than that of the TP group. A fixed effect model was used for data pooling as no significant heterogeneity among included trials was observed.

#### 4.4.2 Subgroup analysis

The subgroup of OS and PFS analysis based on the data of univariate analysis in each included trial showed similar better outcomes in triple therapies of TACE/HAIC TP. Across all subgroups analysis, TACE/HAIC TP was superior to TP for both PFS and OS (**Table 3**). As for PFS, patients without hepatitis (HR: 0.42; 95% CI 0.53–0.73; *P* < 0.05) had significantly better outcomes than those patients without hepatitis (HR: 0.79; 95% CI 0.65–0.98; *P* < 0.05).

**Table 3:** Results of the meta-analysis of OS and PFS.

OS subgroup	No. of trails	HR [95% CI]	P-value	PFS subgroup	No. of trails	HR [95% CI]	P-value
Sex				Sex			
male	4	0.47 [0.36, 0.61]	<0.05	male	3	0.57 [0.44, 0.73]	<0.05
female	4	0.69 [0.38, 1.27]	0.24	female	3	0.49 [0.24, 0.97]	0.23
Hepatitis				Hepatitis			
no	2	0.55 [0.27, 1.14]	0.21	no	1	0.42 [0.53, 0.73]	<0.05
yes	3	0.61 [0.46, 0.80]	<0.05	yes	2	0.79 [0.65, 0.98]	<0.05
Liver cirrhosis				Liver cirrhosis			
no	3	0.44 [0.26, 0.77]	<0.05	no	3	0.46 [0.28, 0.76]	<0.05
yes	3	0.54 [0.40, 0.71]	<0.05	yes	3	0.57 [0.45, 0.73]	<0.05
AFP				AFP			
≤400	4	0.68 [0.45, 1.03]	0.07	≤400	3	0.59 [0.59, 0.60]	<0.05
>400	4	0.44 [0.31, 0.60]	<0.05	>400	3	0.52 [0.39, 0.70]	<0.05
Child-Pugh				Child-Pugh			
A	3	0.48 [0.40, 0.58]	<0.05	A	3	0.54 [0.30, 0.96]	<0.05
B	3	0.43 [0.27, 0.69]	0.27	B	3	0.66 [0.43, 1.02]	0.06
BCLC				BCLC			
B	3	0.61 [0.25, 1.47]	<0.05	B	2	0.63 [0.21, 1.88]	0.41
C	3	0.51 [0.37, 0.70]	<0.05	C	2	0.59 [0.39, 0.91]	<0.05
Extrahepatic metastasis				Extrahepatic metastasis			
yes	4	0.51 [0.36, 0.72]	<0.05	yes	3	0.62 [0.48, 0.80]	<0.05
no	4	0.51 [0.36, 0.72]	<0.05	no	3	0.59 [0.41, 0.84]	<0.05
tumor size				tumor size			
≥10	3	0.50 [0.36, 0.70]	<0.05	≥10	3	0.64 [0.58, 0.72]	<0.05
<10	3	0.49 [0.33, 0.73]	<0.05	<10	3	0.51 [0.30, 0.88]	<0.05
Vascular invasion				Vascular invasion			
no	2	0.47 [0.30, 0.72]	<0.05	no	2	0.57 [0.36, 0.88]	<0.05
yes	2	0.62 [0.39, 0.97]	<0.05	yes	2	0.54 [0.27, 1.07]	0.08

No., number; AFP, Alpha-Fetal Protein.

**Figure 3:**
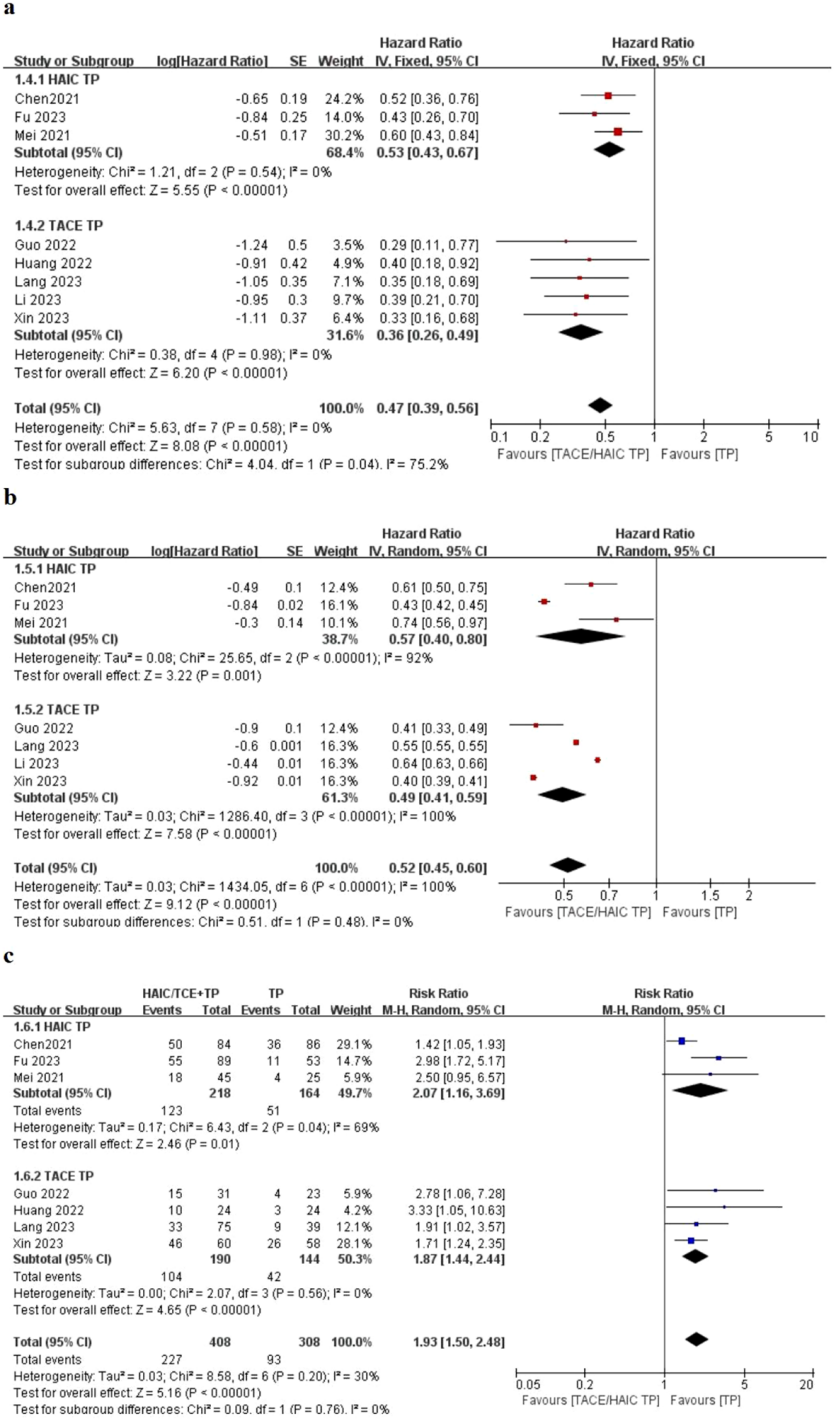
Forest plot about the pooled results of TACE TP versus HIAC TP for unresectable HCC. Outcome: OS **(A)**, PFS **(B)**, and ORR **(C)** in total.

In addition, the subgroup based on chemotherapeutic regime is shown in **Figure 3**. Similar outcomes of OS and PFS indicated that HAIC (HR: 0.53; 95% CI 0.43–0.67; *P* < 0.05 vs. HR: 0.36; 95% CI 0.26–0.49; *P* < 0.05), TACE (HR: 0.57; 95% CI 0.40–0.80; *P* < 0.05 vs. HR: 0.49; 95% CI 0.41–0.59; *P* < 0.05), and ORR (HR: 2.07; 95% CI 1.16–3.69; *P* < 0.05 vs. HR: 1.87; 95% CI 1.44–2.44; *P* < 0.05) were not associated with survival difference in two lines of therapy.

#### 4.4.3 Adverse events

All studies reported treatment-related AEs in the TACE/HAIC TP and TP groups. However, there is significant heterogeneity in the reporting methods and definitions used by the authors of each study, making it difficult and impossible for us to aggregate their results in one statistical method. In all studies, AEs are shown in **Table 4**. The incidence of any grade fever and elevated alanine aminotransferase (ALT) and elevated Aspartate aminotransferase (AST)in TACE/HAIC TP group was higher than that in the TP group (*P* < 0.05). Moreover, the incidence of 3–4 grades elevated AST and platelet decreased in TACE/HAIC TP group was higher than that in the TP group (*P* < 0.05). It is indicated that there was no obvious difference in most AEs between the TACE/HAIC TP group and the TP group.

**Table 4:** Comparison of adverse events between the TACE/HAIC TP group and TP group.

Adverse events						
	Any grades			3/4 grades		
	No. of trails	RR [95% CI]	P-value	No. of trails	RR [95% CI]	P-value
Hypertension	4	1.07 [0.80, 1.42]	0.67	2	1.13 [0.54, 2.35]	0.75
Fever	5	5.56 [1.24, 24.96]	<0.05			
Fatigue	6	1.11 [0.79, 1.57]	0.54			
HFSR	5	0.74 [0.29, 1.87]	0.52	3	1.21 [0.46, 3.17]	0.70
Decreased appetite	6	1.15 [0.80, 1.65]	0.45			
Diarrhea	6	0.83 [0.60, 1.15]	0.27			
Nausea	4	3.30 [1.81, 6.01]	0.19			
Abdominal pain	4	2.26 [1.45, 3.52]	0.26			
Hyperthyroidism	4	7.04 [2.27, 21.83]	0.86			
Hypothyroidism	5	1.01[0.61, 1.68]	0.96			
Elevated ALT	4	1.90 [1.45, 2.50]	<0.05	4	2.47 [0.89, 6.81]	0.08
Elevated AST	4	1.25 [1.07, 1.47]	<0.05	4	2.90 [1.36, 6.17]	<0.05
Leukocytopenia	5	1.38 [0.99, 1.92]	0.33	3	3.86 [0.65,22.71]	0.14
Platelet decreased	5	1.25 [0.96, 1.62]	0.59	4	3.33 [1.22, 9.10]	<0.05
Rash	7	1.22 [0.83, 1.77]	0.31	3	1.38 [0.29, 6.63]	0.69

## 5 Publication bias

Publication bias analysis was not performed for this meta-analysis as the number of included studies was less than 10.

## 6 Discussion

This study is the first systematic meta-analysis aimed at identifying almost all literature studies on the treatment of uHCC with TACE/HAIC TP versus TP, analyzing the efficacy and safety of this comparison and providing a basis for future clinical treatment of uHCC. We conducted a subgroup analysis based on the data of univariate analysis in each included trial to identify beneficial population. The results of this meta-analysis show that the TACE/HAIC TP triple combination regimen led to significantly longer OS, PFS, and better ORR compared with TP, suggesting that the addition of transarterial chemo(embolization) to TKI and PD-1 can prolong survival and improve the prognosis of patients with intermediate or advanced stage HCC. This result was further supported by subgroup analyses, which identified the triple therapy is more effective. The satisfied results may be due to a synergistic antitumor effect of the three treatments.

TACE can embolize tumor blood vessels by injecting iodine oil as well as carrying chemotherapy drugs to continuously kill cancer cells (28). In addition, HAIC refers to the insertion of a percutaneous catheter into the tumor’s blood-supplying artery to inject chemotherapy drugs directly into the liver tumor, which increases the concentration of chemotherapy drugs in tumors and avoids first-pass effects (29). With the advanced diagnosis and high recurrence rate of liver cancer, systematic treatment has become an essential choice and has made effective progress (30). In a phase II trial conducted in China, HAIC showed better efficacy and tolerable toxicity in patients with advanced HCC, which receive better ORR (40.8%) (13). TACE/HAIC involves inserting a catheter into the tumor’s blood supply artery and embolization of the target artery, causing ischemia and necrosis of the tumor tissue. However, arterial embolization cause more serious AEs. Recent evidence suggests that the use of associated liver partition and portal vein alignment (ALPPS) after arterial embolization can rapidly increase the residual volume of the liver in the future. However, due to the high incidence rate and mortality of ALPPS, there is still controversy (31).

Except for the first-line treatment of TKI for uHCC, immunotherapy is constantly breaking through, among which PD-1/PD-L1 is currently a relatively in-depth and thorough ICI in clinical research. PD-1/PD-L1 inhibitors reactivate the body’s immune response to tumor cells by blocking the interaction between PD-1 and PD-L1 (32, 33). Multiple studies have shown that triple therapy is more effective than other combination therapies or single therapy regimens, but there is currently no guidelines recommending the optimal treatment option for uHCC. Although the combination of atezolizumab and bevacizumab is a first-line systemic treatment for uHCC, considering the high cost of the dual immunotherapies and the prevalent risk of gastric bleeding due to cirrhosis, it is crucial to determine the optimal second-line treatment regime. These issues still need to be addressed through future research. Scholars are paying attention to the ongoing III phase of the immunotherapy combination experiment. The IMbrave150 trial showed significantly better OS (not reached vs. 13.2 months), PFS (6.8 vs. 4.3 months), and ORR (33.3% vs. 13.3%) with atezolizumab+bevacizumab than with Sorafenib for uHCC who had received no previous systemic treatment (14). The FDA officially approved atezolizumab+bevacizumab as the first-line treatment for uHCC in 2020, which has stimulated further clinical development of immunotherapy combination approaches for HCC (34). A subsequent randomized, open-label phase 2/3 study conducted in China to compare the efficacy of sintilimab+ IBI305 (a bevacizumab analog) with Sorafenib as a first-line treatment for uHCC, which showed significantly better OS (not reached vs. 10.4 months) and PFS (4.6 vs. 2.8 months) with sintilimab+IBI305 than with Sorafenib for uHCC (35). The CARES-310 trail demonstrated TKI+PD-1 showed a statistically significant and clinically meaningful benefit in PFS (5·6 months vs. 3·7 months) and OS (22·1 months vs. 15·2 months) compared with TKI for patients with uHCC, presenting as a new and effective first-line treatment option for this population (36). And similar outcomes presented that combining TKI and ICI provides an acceptable antitumor efficacy in first-line therapy for advanced-stage HCC patients (37).

The immunotherapy combination therapy is superior to first-line TKI treatment for HCC. The result contributed to several possible reasons as follows: (1) During TKI treatment, changes in immune cell surface antigens may affect the tumor microenvironment and promote tumor escape. Therefore, combination of ICI can restore the immune support microenvironment; (2) targeted anti-angiogenic drugs can restore blood vessel normalization and promote drug release, so a small amount of ICI can be applied to reduce the occurrence of AEs (38, 39).

We conduct this meta-analysis, which showed that TACE/HAIC TP is better than TP for uHCC, and we speculate the mechanism of synergistic anti-tumor effects of triple treatments may be as follows: (1) TKI mainly inhibits activities of vascular endothelial growth factor receptors (VEGFR1-3) and fibroblast and growth factor receptors (FGFR1-4), which the inhibition of VEGFR and FGFR can elicit antitumor immunity and enhance PD-1 checkpoint blockade in HCC (40). (2) Anti-angiogenesis normalizes tumor vessels and breaks the hypoxic microenvironment of tumors, which attenuating the activity of chemoresistance. (3) Tumor necrosis caused by TKIs and chemotherapy regimens can trigger immunogenic cell death, thereby improving the efficacy of immunotherapy (41). (4) The reason why t TACE/HAIC TP is superior to TP is that the chemotherapeutics can have synergistic effects with multiple drugs and effectively reduce the tumor burden (42). For treatment-related AEs, there were no fatal AEs in both the TACE/HAIC TP group and TP group. However, it is still necessary to pay attention to the strong toxic side effects that may be brought about by combined treatment strategies. It is essential to assess the severity of AEs, and immediately discontinue medication, and provide corresponding symptomatic support and treatment if necessary. In addition, based on subgroup analysis of chemotherapy regimens, the results of TACE regime are similar with HAIC regime may be due to the limited number of included trials. Currently, clinical trials of triple therapy based on immune drugs and targeted drugs are increasingly, which not only for the treatment of uHCC but also for patients who may undergo surgical conversion (43, 44). However, more research is needed to explore and reach consensus on the optimal period for transformation, in order to provide patients with reasonable, personalized, and more beneficial treatment regime.

There were several limitations in this meta-analysis. First, selection bias is difficult to avoid as all included studies are retrospective and there is not enough case to analyze as only eight studies were included. Second, considering that all the published studies came from China, the conclusion would not be applicable for the western populations. Last but not least, the diverse of TKI and PD-1 inhibitors affects the consistency of the trails. Additionally, the dosage and duration of HAIC or TACE regimens may cause bias in this study.

## 7 Conclusion

This meta-analysis demonstrated that TACE/HAIC+TKI+PD-1 was superior to TKI+PD-1 with respect to OS, PFS, ORR, and rare AEs for uHCC. Identification of a subgroup for uHCC who may be benefited most from the triple therapy of HAIC+TKI+PD-1 compared to TACE+TKI+PD-1. Further trails need to verify the conclusion.
